# Comparative Genomics and Functional Genomics Analysis in Plants

**DOI:** 10.3390/ijms24076539

**Published:** 2023-03-31

**Authors:** Jiacheng Wang, Yaojia Chen, Quan Zou

**Affiliations:** 1 Institute of Fundamental and Frontier Sciences, University of Electronic Science and Technology of China, Chengdu 610054, China; 2 Yangtze Delta Region Institute (Quzhou), University of Electronic Science and Technology of China, Quzhou 324003, China

Comparative genomics and functional genomics are two basic branches of plant genomics. They play a crucial role in our understanding of the mechanisms of plant evolution and adaptation. Plant genomes are relatively large and complex; therefore, the comparative and functional analysis of plant genomes is a challenging but important task. With an explosion in the number of genome projects due to advancements in sequencing technologies, particularly the next-generation sequencing methods in the late 2000s, this field has become more sophisticated, making it possible to deal with many genomes in a single study. To advance research in the field of plant genomics, we are pleased to organize this Special Issue in *The International Journal of Molecular Sciences* entitled “Comparative Genomics and Functional Genomics Analysis in Plants”.

Specifically, this Special Issue contains 21 papers that cover various aspects of plant genomics and functional genomics from diverse species, including cotton, wheat, legume, cultivated peanuts, bermudagrass, peach fruit, kiwifruit, *Prunus mume*, *Heracleum moellendorffii* Hance, Siberian larch, Juglandaceae species, rice, mulberry, garlic, shrub willow, and cucumber. Some of these papers focused on the structure and evolution of plant genomes and studied the evolutionary history and diversity of plant genomes. Other papers focused on plant gene expression and regulation, such as transcriptomics and epigenetics, and plant metabolomics and proteomics. In addition, several papers explored functional genomics of plant genomes, such as gene functional annotation and the development of functional genomics tools. According to their specific research issues, the twenty-one papers are summarized as follows.

Research on crop stress resistance is an important topic of this Special Issue. Seven papers included in this section focused on crop resistance or response to abiotic/biotic stresses. For example, Wei et al.(2022) [1] performed extensive comparative analyses in cotton (*Gossypium* spp.) and identified many cis-elements and key gene pathways for resistance to abiotic stresses. The weighted gene co-expression network analysis (WGCNA) further demonstrated a good correlation between the GhSCL-8 gene and salt tolerance. Another abiotic stress capable of significantly impacting plants is low temperature. The comparative genome and RNA-seq data analysis of eight rosaceae genomes revealed the significant response of PmHDACs genes to cold stress in *Prunus mume* [2]. Liu et al. (2022) [3] applied a controlled atmosphere (CA) treatment to peach fruit and explored its effects on alleviating a chilling injury and maintaining the fruit flavor quality. They found that the CA elevated the sucrose content and the degree of fatty acid unsaturation under cold storage, which presented clues for clarifying the mechanism of resistance to cold stress. Li et al. (2022) [4] demonstrated the function of the TaKNOX14-D gene under conditions of mechanical injury and cold stress by performing a genome-wide identification and a gene expression and functional analysis in wheat, providing a better understanding of the role of TAKNOX in abiotic stress. As the only species in the pine family (Pinaceae) with a seasonal deciduousness, Siberian larch is of interest to some researchers. Using a comparative analysis of the genomes of evergreen and deciduous trees, Batalova et al. (2022) [5] found that the genes that control the EXL2 and DRM1 proteins, which are associated with leaf senescence, are most represented in Siberian larch. At the same time, the effects of biotic stress on plant growth patterns are also of interest to some researchers. Wang et al. (2022) [6] performed a bioinformatics and expression pattern analysis of six MnASIs on transcriptome data from mulberry (*Morus notabilis*) and confirmed that MnASI1 was involved in the defense response to Botrytis cinereal infection. On the other hand, Czyż et al. (2022) [7] investigated the nitrogen-fixing symbiosis mechanism between legumes with Rhizobia. Specifically, the experimental results indicated that losses of the genes involved in trichome development, defense, and wounding responses were strongly associated with rhizobial symbiosis in legumes.

Five papers included in the second portion are focused on plant growth and development. By studying gene expression data from upland cotton, Wang et al. (2022) [8] identified a key factor in the growth and development of wrinkled cotton leaves and demonstrated that this process may be regulated by plant hormone signals. Gan et al. (2022) [9] performed a genome-wide association study (GWAS) and a WGCNA of the genetic diversity of 91 wild bermudagrass resources. They identified genes closely related to several traits, including plant height and IAA content, which uncovered the breeding selection history of different plant types from diverse bermudagrass. Luo et al. (2022) [10] performed a genome-wide analysis of *Actinidia chinensis* and *A. eriantha* and identified key genes that regulate chlorophyll A content, providing evidence for the characteristics and evolutionary patterns of the LHC gene in kiwifruit. Zhang et al. (2022) [11] analyzed the GRF gene families in whole genome of four juglandaceae species and identified several genes related to the development of walnut embryos and male and female flower buds, providing guidance for the future study of GRF gene families in walnuts. Cao et al. (2022) [12] identified several genes related to the growth process of cotton plants by performing functional genome studies on putative m6A methyltransferase in cotton, which laid a foundation for improving the agronomic traits of cotton.

Flowering and breeding are also important fields in plant genomics. Six papers included in this section have conducted extensive research on these issues, proving that this field has an important role to play in solving the problem of global plant reproduction. Hyden et al. (2023) [13] presented de novo assemblies and annotations of 11 shrub willow genomes and found that key sex determination genes are missing in *S. viminalis* and *S. koriyanagi*. They further hypothesized that a unique sex determination system exists in these species that differs from Populus and other Salix species. Shemesh-Mayer et al. (2022) [14] presented conjoint genomic and transcriptome analyses of the regulatory network in flowering garlic genotypes and proposed that the process of fertility deprivation in garlic cultivars is based on the loss of the transcriptional functions of the specific genes. Rice and wheat are two of the most important food crops in the world. Mun et al. (2022) [15] investigated the regulatory mechanism of grain breakage in rice and reported a new gene involved in seed breakage, providing new clues for improving rice yield. Meanwhile, Fang et al. (2022) [16] identified several key variety-specific hub transcript factors by analyzing wheat transcriptome data and gene co-expression networks, providing breeders with a list of potential targets and clues. Flowering time has a strong impact on biomass production, and it is therefore necessary to understand how flowering is regulated in important crops. Zhong et al. (2022) [17] explored the evolutionary characteristics of genes that regulate flowering time in cultivated peanuts through comparative genomics and further identified their floral induction and mechanisms with genotypes, photoperiod, and temperature. Using genome-wide classification and functional annotation, Cenci et al. (2022) [18] effectively characterized a large gene family across multiple species and established a classification framework for gene functional transfer, enabling a better understanding of the evolutionary history of one gene.

Three papers have demonstrated a strong interest in the study of the genomes of plants and their association with some important traits. Sathasivam et al. (2022) [19] discussed the expression mechanism of the carotenoid pathway in *Heracleum moellendorffii* Hance and analyzed the expression distribution of related genes in different organs, enabling the investigation of strategies that could enhance the anti-carcinogenic properties of *H. moellendorffii*. Kim et al. (2023) [20] proposed a combined approach based on single-nucleotide polymorphism (SNP) array and GWAS analyses and unraveled the genetic basis of complex root traits in soybean, which will provide valuable clues for further genetic research and the breeding of climate-adaptable soybeans with improved root traits. By integrating biological and bioinformatics data, Turek et al. (2023) [21] performed a genome assembly on the B10v3 cucumber genome and presented a well-organized and annotated reference genome of cucumber, contributing to better understanding of the cucumber genome.

Overall, the 21 papers in this Special Issue showcase recent research advances in the fields of comparative genomics and functional genomics in plants, covering a wide range of topics and providing important insights into the basic biology, growth, and development of plants. We believe that this Special Issue will be a useful resource for researchers in the field of plant comparative and functional genomics. It will also provide important clues for a better understanding of plant biological processes and gene regulation, as well as new strategies and methods for plant development and breeding. We hope that the publication of this Special Issue will encourage more researchers and scholars to devote themselves to the study of plant comparative and functional genomics and promote the development of this field.

Finally, we would like to thank *The International Journal of Molecular Sciences* for providing us with a platform to display these research results. We would also like to thank all contributors for their support and contributions, as well as this Special Issue’s panel of reviewers for their hard work and valuable input. We hope that this Special Issue will receive widespread attention and make further contributions to the research in the field of plant comparative and functional genomics.
